# Optimization of Glutamine Peptide Production from Soybean Meal and Analysis of Molecular Weight Distribution of Hydrolysates

**DOI:** 10.3390/ijms13067483

**Published:** 2012-06-18

**Authors:** Yanli Xie, Xinhong Liang, Min Wei, Wenhong Zhao, Baoshan He, Qiyu Lu, Quangong Huo, Chengye Ma

**Affiliations:** 1 School of Food Science and Technology, Henan University of Technology, Zhengzhou 450001, China; E-Mails: wei_min80@163.com (M.W.); zhwh2008@yahoo.cn (W.Z.); hebaoshan@126.com (B.H.); qiyulu7120@vip.sina.com (Q.L.); huo_qg@126.com (Q.H.); hnmcy@sina.com (C.M.); 2 School of Food Science and Technology, Henan Institute of Science and Technology, Xinxiang 453003, China; E-Mail: liangxinhong2005@163.com

**Keywords:** glutamine peptide, soybean meal, enzymatic hydrolysis, response surface methodology, molecular weight distribution

## Abstract

The process parameters of enzymatic hydrolysis and molecular weight distribution of glutamine (Gln) peptides from soybean meal were investigated. The Protamex^®^ hydrolysis pH of 6.10, temperature of 56.78 °C, enzyme to substrate ratio (E/S) of 1.90 and hydrolysis time of 10.72 h were found to be the optimal conditions by response surface methodology (RSM) for a maximal degree of hydrolysis (DH) value of 16.63% and Gln peptides content at 5.95 mmol/L. The soybean meal was hydrolyzed by a combination of Protamex^®^ and trypsinase so that DH and Gln peptides would reach 22.02% and 6.05 mmol/mL, respectively. The results of size exclusion chromatography indicated that the relative proportion of the molecular weight <1000 Da fraction increased with DH values from 6.76%, 11.13%, 17.89% to 22.02%, most notably the 132–500 Da fractions of hydrolysates were 42.14%, 46.57%, 58.44% and 69.65%. High DH values did not lead to high Gln peptides content of the hydrolysate but to the low molecular weight Gln peptides.

## 1. Introduction

Soybean is one of the most important oilseeds in the world. The industrial processing of soybean yields two products, oil and a low-valued defatted soybean meal. Soybean meal protein has shown markedly higher values and is good source of bioactive peptides [1,2]. Glutamine is the most abundant amino acid in the human body, where it plays a number of important physiological roles that fuel the rapid proliferation of cells (fibroblasts, immune and gastrointestinal cells) [3]. The glutamic acid/glutamine content in wheat, corn and soybean meal is 31.90%, 20.48% and 18.82%, respectively [4–6]. Glutamine deficiency may compromise many important cellular protective, immunosuppressive and disease recovery processes that affect patient survival [7]. Prabhu reported that oral supplementation of glutamine or glutamic acid altered the brush border membrane in the intestine to prevent postoperative complications after surgical manipulation [8].

Recent studies suggest that plasma glutamine levels are a useful indicator of immunocompetence and overtraining syndrome and assert that glutamine is a potentially useful dietary supplement for subjects engaging in intensive athletic training [9]. However, the solubility and stability of free glutamine were found to be limited, and it is easily broke down into pyroglutamic acid and ammonia during autoclaving [10], which rules out incorporation into currently available nutritional preparations. Following this, the dipeptide concept with stable, highly soluble synthetic glutamine was investigated to overcome these drawbacks. Peptide-bound glutamine extracted from plant protein by enzymatic hydrolysis has the advantage of good safety in enteral nutrition. Wheat gluten treated with protease was developed to produce a peptide-bound glutamine for enteral nutrition and the oligopeptide fraction enhanced mucosal protein content significantly more effectively in fasting rats when compared to a simulated free amino acid mixture. Additionally, rats showed greater resistance to methotrexate-induced enterocolitis when they were fed the oligopeptide fraction rather than a simulated free amino acid mixture [11]. Dietary supplemented glutamine and soybean peptides have important effects on the structure of the kidney filtration barrier and the content of total protein, albumin and 2-microglobulin in the urine of an over-training group [12]. Plant protein hydrolysates were mainly used as protein ingredients or supplements in food or as ingredients in special formulations for clinical nutrition [13]. Molecular weight plays an important role in the functional and biological properties of peptides [14], and therefore, it is essential to determine molecular weight distribution of protein hydrolysates and the process parameters that control it. This research aimed to optimize glutamine peptide production from soybean meal and to analyze the molecular weight distribution of hydrolysates, since they promise better utilization of soybean by-products from extracting oil.

## 2. Results and Discussion

### 2.1. Statistical Analysis and Model Fit of Hydrolysis Process

The central composite design of the four independent variables (pH, temperature, E/S and reaction time) and the responses (DH value and Gln content) are shown in Table 1. Multiple regression analysis was performed on the experimental data to obtain the predictive model using the following second-order polynomial Equations (1,2):

(1) $$\begin{array}{l} {\text{R}}_{1}:\text{DH}=15.257-0.795\;{\text{X}}_{1}+0.118\;{\text{X}}_{2}+0.651\;{\text{X}}_{3}+0.379\;{\text{X}}_{4}-0.589\;{\text{X}}_{1}{\text{X}}_{2}+0.197\;{\text{X}}_{1}{\text{X}}_{3}-0.048\\ {\text{X}}_{1}{\text{X}}_{4}-0.227\;{\text{X}}_{2}{\text{X}}_{3}-0.092\;{\text{X}}_{2}{\text{X}}_{4}-0.013\;{\text{X}}_{3}{\text{X}}_{4}-0.313\;{{\text{X}}_{1}}^{2}-0.359\;{{\text{X}}_{2}}^{2}-0.200\;{{\text{X}}_{3}}^{2}-0.139\;{{\text{X}}_{4}}^{2} \end{array}$$

(2) $$\begin{array}{l} {\text{R}}_{2}:\text{Glncontent}=5.647-0.075\;{\text{X}}_{1}-0.006\;{\text{X}}_{2}+0.066\;{\text{X}}_{3}+0.085\;{\text{X}}_{4}+0.002\;{\text{X}}_{1}{\text{X}}_{2}+0.111\;{\text{X}}_{1}{\text{X}}_{3}-\\ 0.053\;{\text{X}}_{1}{\text{X}}_{4}+0.004\;{\text{X}}_{2}{\text{X}}_{3}-0.004\;{\text{X}}_{2}{\text{X}}_{4}+0.079\;{\text{X}}_{3}{\text{X}}_{4}-0.062\;{{\text{X}}_{1}}^{2}-0.019\;{{\text{X}}_{2}}^{2}+0.001\;{{\text{X}}_{3}}^{2}+0.026\;{{\text{X}}_{4}}^{2} \end{array}$$

**Figure 1:**
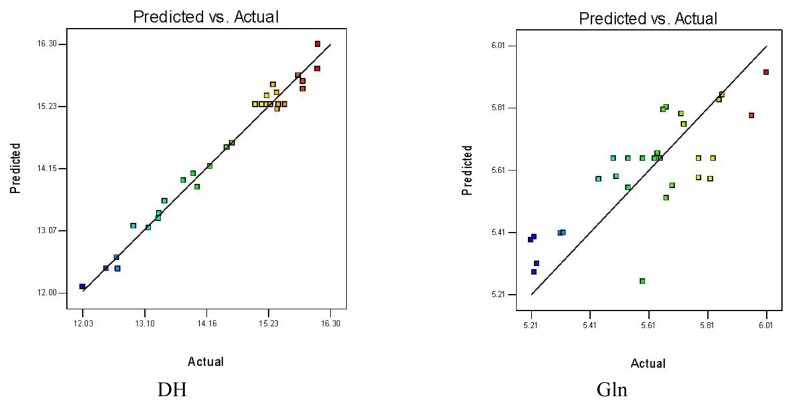
Predicted response *vs.* actual value for degree of hydrolysis (DH) and glutamine (Gln) content.

Significance of the coefficients to DH was determined using the F-test and p-value for the influence of pH, temperature, E/S and reaction time (Table 2). The ANOVA of the quadratic regression model shows high significance for the model (*p* < 0.0001) and it is therefore suitable for monitoring optimization. The effect of pH, E/S and reaction time exerted highly significant effect on DH values (*p* < 0.0001), and hydrolysis temperature was also significant (*p* < 0.05). Moreover, the quadratic term of pH (X_1_^2^), temperature (X_2_^2^), E/S (X_3_^2^) and reaction time (X_4_^2^) also showed highly significant effects on DH values (*p* < 0.001). In addition, two-way interaction for parameters was significant for pH and temperature (*p* < 0.0001), pH and E/S, and temperature and E/S (*p* < 0.001), and not significant for interaction between pH and reaction time, temperature and reaction time or E/S and reaction time (*p* > 0.05). The statistical analysis for the model (Table 2) showed the lack of fit was not significant for pure error (*p* > 0.05). The coefficient of determination R^2^ was 0.9818 and Adj *R*^2^ was 0.9649, *i.e.*, the model fit could explain 96.49% of total variability within the range of values studied. In further analysis, each observed value for DH (Y_1_′) was compared with the adequate predicted value (Y_1_) (Figure 1A). All these results imply that the model gives a satisfactory mathematical description of the hydrolysis process Equation (1).

The ANOVA of the quadratic regression model gives no significance for the Gln content model (*p* > 0.05) (Table 2). These variables had no significant effect on response (*p* > 0.05). Given *R*^2^ at 0.6255 and Adj *R*^2^ at 0.2760, the model gave a poor fit with the experimental data and a disappointing mathematical description for Gln yield. Figure 1B shows that the actual gap between Gln response (Y_2_) values and the predicted values (Y_2_′). In general, peptides activity is affected considerably by the DH of the protein substrate(s), and the independent variables (pH, temperature, E/S and reaction time) have an effect on the DH. The high peptides activity was not only a result of extensive hydrolysis, but also due to some intrinsic properties of the protein substrate(s) or specificity of the enzyme(s) [15]. The Gln peptides content was not observed to change obviously with increasing DH, so the Gln content was not appropriate as an objective function. The molecular weight of peptides greatly affects their functional and biological properties and the low molecular weight of Gln peptide results in faster absorption inside the body, so the activity of peptide segments were affected by the DH value to some extent [2,14].

### 2.2. Effect of pH, Temperature, E/S Ratio and Reaction Time on Hydrolysis

Response surface methodology (RSM) is an effective, widely accepted method of solving multivariate problems to properly predict the values of the response variables in many types of research [16]. It generates 3D response surface and 2D contour plots to show the interrelationships between two tested variables and the relationship between responses and experimental levels of each variable. The optimal values of the selected variables were obtained by regression analysis on Design-Expert 7.0. Two variables within the experimental range are depicted with the third variable kept constant at zero. Different shapes of contour plots indicate different interactions between two variables and circular plots of response surfaces suggest negligible interaction between corresponding variables while elliptical or saddle-shaped plots highlight significant interaction between corresponding variables. For saddle contour plots, the optimum values are obtained at the point of intersection of lines formed by joining the locus [17].

**Figure 2:**
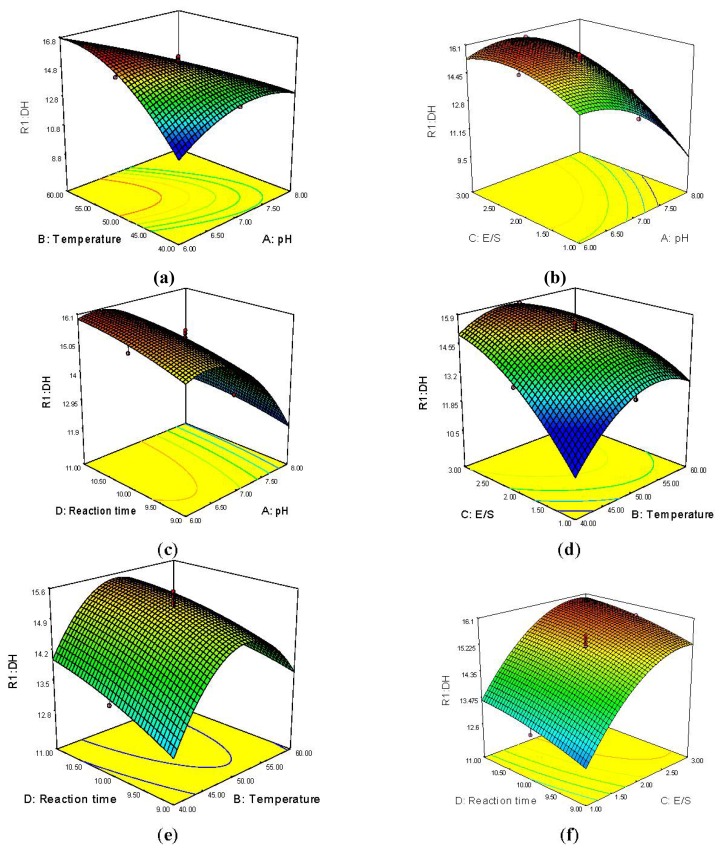
Response surface plots and contour plots for the interactive effects of variables on the DH value. (**a**) pH and temperature; (**b**) pH and E/S ratio; (**c**) pH and reaction time; (**d**) temperature and E/S ratio; (**e**) temperature and reaction time; (**f**) E/S ratio and reaction time.

The 3D surface and 2D contour plots (Figure 2) were drawn to illustrate the principal interactive effects of the independent variables on the dependent DH. Figure 2A shows the 3D surface and contour plots of the effect of pH and temperature on DH. Visibly, DH increased with pH until a peak at about 7.0. There was an ellipse in the contour plot for DH expressed as a function of pH and temperature, which indicates the significance of interactions between pH and temperature (Figure 2A). In the E/S range of 1.0%–3.0%, considered as the economical usage range of the enzyme, the DH value increased with the E/S and peaked at about 2.5% (Figure 2B). The saddle contour plots in Figure 2B plainly show that interactions between pH and E/S ratio also reached their optimum levels. DH barely increased with reaction time from 9 to 11 h as seen in Figure 2C. Low pH favored the higher DH value, which dropped linearly as pH increased from 6.3 to 8.0. The circular contour plots of response surfaces suggest that the interaction of pH and reaction time is negligible with the corresponding variables (Figure 2C). Figure 2D shows that DH increased gradually as temperature rose in the medium until gradually peaking at about 49 °C. DH also tended to increase along with E/S. The elliptical contour plots indicate the significance of the interaction between temperature and E/S (Figure 2D). As shown in Figure 2E, DH increased until the temperature reached an optimal peak at about 50 °C. In terms of reaction time, DH slowly increased as hydrolysis was prolonged from 9 to 11 h. Circular contour plots, which signal non-significance for the interaction between temperature and reaction time, were clearly shown. Further, Figure 2F shows that DH increased along with E/S from 1% up to 2.8% and peaked at about 2.8%. Likewise, DH rose slightly as reaction time increased from 9 to 11 h. The plot in Figure 2F for DH as a function of E/S and reaction time was circular, thereby illustrating that this interaction was not significant.

### 2.3. Optimization and Validation of the Experimental Design

Optimal conditions of the variables were pH 6.10, 56.78 °C, E/S 1.90 and a reaction time of 10.72 h as determined on Design-Expert, under which the predicted DH value was 16.78%. Applying these conditions, the experiment scored 16.63% while Gln content was 5.95 mmol/L. These two percentages are close enough to validate the model. The optimum parameters of trypsinase previously reported by our laboratory were: pH 7.83, 50 °C, E/S 4.5 and hydrolysis time 3.7 h such that the DH value and Gln could reach 12.79% with glutamine content at 5.92 mmol/mL [18]. The defatted soybean meal was hydrolyzed by trypsinase and Protamex^®^ at their respective optimal parameters such that DH and Gln reached 22.02% and 6.05 mmol/mL, respectively, so the soy meal was well hydrolyzed. The diversification of hydrolysis degree and molecular weight distribution were investigated during the process of defatted soybean meal hydrolysis.

### 2.4. Effect of the Diversification of Hydrolysis Degree on the Molecular Weight Distribution

**Figure 3:**
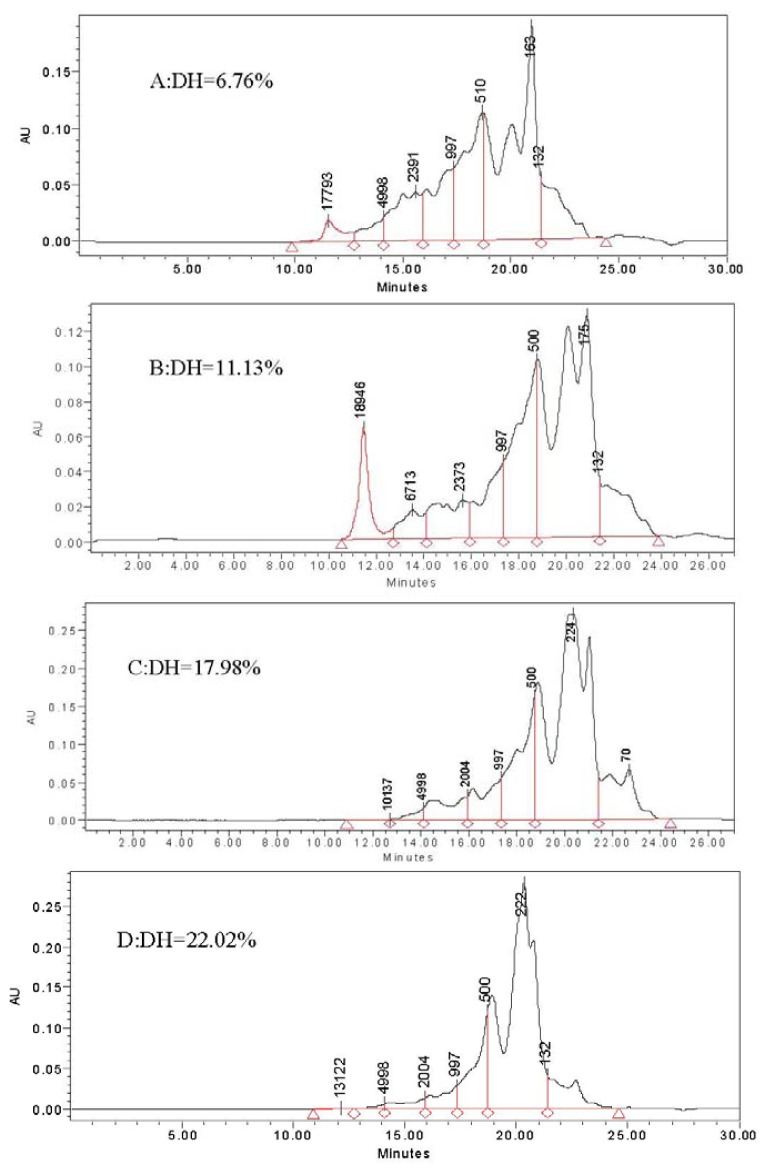
Effect of DH on the molecular weight distribution of hydrolysates. (**A**): DH = 6.76%; (**B**): DH = 11.13%; (**C**): DH = 17.98%; (**D**): DH = 22.02%.

The molecular weight distribution profiles of the defatted soybean meal hydrolysates obtained with trypsinase and Protamex^®^ are presented in Figure 3. The molecular weight distribution of defatted soybean meal hydrolysates ranged from 100 to 10,000 Da and most soybean meal peptides were smaller than 1000 Da (Figure 3). According to their molecular weight ranges: >10,000, 10,000–5000, 5000–2000, 2000–1000, 1000–500, 500–132 and <132, the seven absorption peaks in the chromatogram were marked out. Comparing the relative areas, there could be some differences in interpretation of the areas due to the lack of the molar extinction coefficients required to do an accurate quantitation. The surface area of the >1000 Da group decreased while that of <1000 Da groups increased using four DH values ranging from 6.76%, 11.13%, 17.89% and 22.02%, respectively. The relative proportions of molecular weight <1000 Da fraction of the hydrolysates were 71.96%, 74.55%, 86.52% and 92.37% at DH values of 6.76%, 11.13%, 17.89% and 22.02%, respectively. The relative proportions for 132–500 Da fraction of hydrolysates were 42.14%, 46.57%, 58.44% and 69.65% with DH values of 6.76%, 11.13%, 17.89% and 22.02%, respectively. The results indicate that defatted soybean meal hydrolysates contained many short peptides, greatly due to trypsinase and Protamex^®^. The functional and biological properties of protein hydrolysates correlated with the degree of hydrolysis and molecular weight [14,19].

## 3. Experimental Section

### 3.1. Materials

Defatted soybean meal was supplied by Xuchang Bangdi Protein Industry Co., Ltd. (Henan, China). Protamex^®^ (40,000 U/g) and trypsinase (48,000 U/g) commercial enzymes were kindly donated by Novozyme (Beijing, China). L-Gly-Gln standards were purchased from Sigma Chemical Co. (St. Louis, MO, USA). All other reagents used were of analytical grade. [Bis(trifluoroacetoxy)iodo]benzene was purchased from Shanghai Yurlic Chemical S&T Co.Ltd (Shanghai, China). Isothiocyanic acid phenyl ester was purchased from Acros Organics (Pittsburgh, USA). Acetonitrile (chromatographically) was purchased from Tianjing Kemiou chemreagen Co.Ltd (Tianjing, China). Ethylic acid, natrium aceticum, pyridine, triethylamine, trifluoroacetic acid (TFA) and formaldehyde were of analytical-grade.

### 3.2. Enzymatic Hydrolysis

The defatted soybean meal concentration was fixed at 12% (w/v) and hydrolyzed with Protamex^®^ according to the hydrolysis conditions defined by the experimental design. Hydrolysis pH was maintained at the desired value by continuous addition of 0.1 N NaOH and 0.1 N HCl. After the required digestion time, the reaction was stopped by heating the solution to 80 °C for 20 min to deactivate the enzyme. DH and Gln content were determined from the supernatant after centrifugation at 4000× g for 15 min [20].

### 3.3. Determination of the Degree of Hydrolysis

DH was the percentage of cleaved peptide bonds (*h*) out of total number of such bonds in the substrate (*h*_tot_) and was calculated from the amount of base consumed as given below (Equation (3)) [2,21]:

(3) $$\text{DH}\%=\frac{h\times 100}{h_{\text{tot}}}=\frac{B\times N_{\text{b}}\times 100}{\alpha \times M_{\text{p}}\times h_{\text{tot}}}$$

where *B* is base consumption in mL; *N*_b_ is normality of the base; α is average degree of dissociation of the α-NH_2_ groups; *M*_p_ is mass of protein (*N* × 6.25) in g; *h* is the hydrolysis equivalents in meqv/g protein and *h*_tot_ is total number of peptide bonds in the protein substrate (7.75 meqv/g soy protein).

### 3.4. Quantitative Analysis of Glutamine in Soybean Meal Hydrolysates

Conversion of glutamine residues into diaminobutyric acid (DABA) was performed by adding 100 μL bis-1,1-trifluoroacetoxy-iodobenzene (BTI) into acetonitrile (10 mg/mL), and 25 μL aqueous pyridine (50 μmol/mL) was added into 100 μL hydrolysis protein solution and incubated at 50 °C for 20 h. This prehydrolytic reaction was generated for glutamine residues with BTI and converted glutamine to the corresponding diaminobutyric acid (DABA) and was then dried immediately by vacuum.

Acid hydrolysis was performed by adding 200 μL of 6 M HCl into 200 μL DABA sample. The obtained solutions were purged with nitrogen gas for 2 min, then hydrolysis was carried out at 110 °C for 23 h. The acid digested samples were freeze dried.

HPLC analysis was performed on a Waters 2695 Alliance System (Waters Corporation, Millipore, Milford, MA, USA), UV detector and a Waters symmetry column C18 oven set to 40 °C, flow rate 0.3 mL/min and injection volume 5 μL. The freeze dried acid digested samples was dissolved in mobile phase and loaded on a 2.1 × 50 mm C18 column (Waters Corporation, Millipore, Milford, MA, USA), then separated and eluted using an aqueous sodium acetate-acetonitrile gradient and detected at 254 nm [22].

### 3.5. Size Exclusion Chromatography

Molecular weight distribution of peptides in the different hydrolysates was determined by gel permeation chromatography (TSKgel 2000 SWXL 300 mm × 7.8 mm) as described by Dong [23]. The sample was dissolved in mobile phase and filtered by micropore membrane and the injection volume was 20 μL. The liquid chromatographic system consisted of a Waters 600 automated gradient controller pump (Waters Corporation, Millipore, Milford, MA, USA) and a 2487 UV detector at 220 nm. The mobile phase_isocratic elution consisted of 0.1% (v/v) TFA and acetonitrile (55:45). The flow rate was 0.5 mL/min. System control and data processing was performed using Empower GPC software (Version 2.0, Waters Corporation, Millipore: Milford, MA, USA). A molecular weight calibration curve was prepared from the elution time of the peak volume using five standards: Cytochrome C (12,500 Da), aprotinin (6500 Da), bacitracin (1450 Da), Gly-Gly-Tyr-Arg (451 Da) and Gly-Gly-Gly (189 Da). A relationship between the retention time and the log of the molecular mass of peptides used as standards was established. In the different hydrolysates, peptides were sorted in 5 fractions covering the ranges of 0–500 Da (fraction V), 500–1000 Da (fraction IV), 1000–2000 Da (fraction III), 2000–5000 Da (fraction II) and above 5000 Da (fraction I). The relative areas of each fraction were given in percent of the total area.

### 3.6. Experiment Design

Temperature (X_1_), pH (X_2_), E/S (X_3_) and reaction time (X_4_) were chosen as independent variables and optimized using a central composite rotatable design (CCRD) [13]. Four key independent variables at five levels were retained and individually coded as −2, −1, 0, +1, +2 (Table 3). The parameters and their ranges were chosen on the basis of the preliminary experimentation data not shown. All experiments were done in quadruplicate, results were averaged and are presented as Y_1_ and Y_2_ for DH and Gln, respectively. The behaviour of the system is explained by the following Equation (4):

(4) $$y=\beta_{0}+\sum_{i=1}^{3}\beta_{i}\;x_{i}+\sum_{i=1}^{3}\beta_{ii}\;x_{i}^{2}+\sum_{i=1}^{2}\sum_{j=i+1}^{3}\beta_{ii}\;x_{i}x_{j}$$

where y is the dependent variable (DH value and Gln content); β_0_ is a constant; β*_i_*, β*_ii_* and β*_ij_* are coefficients estimated by the model; and *x_i_*, and *x_j_* are levels of the independent variables. They represent the linear, quadratic and cross product effects of the X_1_, X_2_, X_3_ and X_4_ factors respectively.

### 3.7. Statistical Analysis

Design Expert 7.0 statistical software (Stat-Ease Inc., Minneapolis, MO, USA) was used to analyze the experiment design. One-way variance analysis (ANOVA) (*p* < 0.05) was conducted for the response values obtained by the RSM model.

## 4. Conclusions

The effects of four independent variables on the production of glutamine peptides from soybean meal were determined using response surface methodology as a predictive tool. The parameters of the DH model were estimated by multiple linear regression, obtaining a good fit with the experimental data, given that the Adj *R*^2^ was 0.9649. With a combination of Protamex^®^ and trypsinase, DH values and Gln peptides content were 22.02% and 6.05 mmol/mL, respectively. At DH values of 6.76%, 11.13%, 17.89% and 22.02%, the relative proportions of molecular weight <1000 Da fraction of hydrolysates were 71.96%, 74.55%, 86.52% and 92.37%; more notably the 132–500 Da fractions were 42.14%, 46.57%, 58.44% and 69.65%. The hydrolysates of defatted soybean meal contained many short peptides and were greatly degraded by trypsinase and Protamex^®^.

## Figures and Tables

**Table 1:** Four-factor central composite design and values of responses. Independent variables: Temperature (X_1_), pH (X_2_), E/S (X_3_) and reaction time (X_4_).

	Independent Variable				Response	
		
	X_1_	X_2_/°C	X_3_/%	X_4_/h	Y_1_:DH, %	Y_2_:Gln mmol/L
1	−1	1	1	−1	15.83 ± 0.18	5.32 ± 0.06
2	1	−1	−1	1	13.34 ± 0.20	5.23 ± 0.15
3	−1	−1	−1	1	14.23 ± 0.26	5.72 ± 0.14
4	−1	−1	1	−1	14.52 ± 0.14	5.31 ± 0.06
5	−2	0	0	0	15.32 ± 0.13	5.54 ± 0.16
6	0	0	0	0	15.41 ± 0.06	5.49 ± 0.21
7	0	2	0	0	13.94 ± 0.14	5.69 ± 0.06
8	−1	−1	−1	−1	13.17 ± 0.25	5.64 ± 0.04
9	0	0	−2	0	12.91 ± 0.02	5.67 ± 0.08
10	1	1	1	1	14.01 ± 0.16	5.66 ± 0.11
11	−1	1	−1	1	16.08 ± 0.23	5.73 ± 0.07
12	0	0	2	0	15.75 ± 0.09	5.96 ± 0.10
13	0	0	0	0	15.27 ± 0.30	5.78 ± 0.14
14	0	0	0	0	15.21 ± 0.13	5.59 ± 0.08
15	1	1	−1	−1	12.03 ± 0.28	5.21 ± 0.04
16	1	1	−1	1	12.62 ± 0.12	5.22 ± 0.12
17	0	0	0	0	15.01 ± 0.10	5.54 ± 0.19
18	−1	1	−1	−1	15.39 ± 0.23	5.63 ± 0.11
19	0	0	0	2	15.38 ± 0.22	6.01 ± 0.18
20	0	0	0	−2	13.77 ± 0.11	5.82 ± 0.15
21	1	−1	1	−1	14.61 ± 0.09	5.44 ± 0.06
22	−1	−1	1	1	15.83 ± 0.06	5.86 ± 0.13
23	−1	1	1	1	16.09 ± 0.19	5.85 ± 0.05
24	1	1	1	−1	13.35 ± 0.21	5.50 ± 0.11
25	0	0	0	0	15.52 ± 0.19	5.65 ± 0.09
26	0	−2	0	0	13.45 ± 0.22	5.78 ± 0.11
27	1	−1	1	1	15.21 ± 0.13	5.67 ± 0.08
28	1	−1	−1	−1	12.64 ± 0.17	5.22 ± 0.12
29	2	0	0	0	12.44 ± 0.22	5.59 ± 0.21
30	0	0	0	0	15.12 ± 0.21	5.83 ± 0.15

**Table 2:** Significance of regression coefficients for degree of hydrolysis (DH) and glutamine (Gln) content.

Source	Sum of Squares		df	Mean Square		*F*-Value		*p*-Value/Prob > *F*	
				
	DH	Gln		DH	Gln	DH	Gln	DH	Gln
Model	42.196	0.905	14	3.014	0.065	57.952	1.790	<0.0001 ****	0.1377 ns
Linear									
X_1_	15.185	0.137	1	15.185	0.137	291.963	3.780	<0.0001 ****	0.0709 ns
X_2_	0.334	0.001	1	0.334	0.001	6.416	0.026	0.0230 *	0.8741 ns
X_3_	10.179	0.105	1	10.179	0.105	195.719	2.917	<0.0001 ****	0.1083 ns
X_4_	3.443	0.175	1	3.443	0.175	66.198	4.849	< 0.0001 ****	0.0437 ns
Quadratic									
X_1_^2^	2.680	0.103	1	2.680	0.103	51.537	2.840	<0.0001 ****	0.1126 ns
X_2_^2^	3.532	0.010	1	3.532	0.010	67.915	0.264	<0.0001 ****	0.6148 ns
X_3_^2^	1.098	0.000	1	1.098	0.000	21.118	0.001	0.0004 ***	0.9707 ns
X_4_^2^	0.529	0.019	1	0.529	0.019	10.168	0.528	0.0061 ***	0.4788 ns
Interaction									
X_1_X_2_	5.558	0.000	1	5.558	0.000	106.864	0.002	<0.0001 ****	0.9690 ns
X_1_X_3_	0.620	0.196	1	0.620	0.196	11.924	5.422	0.0035 **	0.0343 ns
X_1_X_4_	0.037	0.045	1	0.037	0.045	0.7125	1.251	0.4119 ns	0.2810 ns
X_2_X_3_	0.824	0.000	1	0.824	0.000	15.835	0.008	0.0012 **	0.9278 ns
X_2_X_4_	0.135	0.000	1	0.135	0.000	2.597	0.008	0.1279 ns	0.9278 ns
X_3_X_4_	0.003	0.101	1	0.003	0.101	0.053	2.792	0.8210 ns	0.1155 ns
Statistic analysis for the model									
Residual	0.780		15	0.052	0.036				
Lack of Fit	0.605		10	0.061	0.045	1.732	2.491	0.2829 ns	0.1627 ns
Pure Error	0.175		5	0.035	0.018				
Cor Total	42.976		29						

* Significant at *p* < 0.05;

** Significant at *p* < 0.01;

*** Significant at *p* < 0.001;

**** Significant at *p* < 0.0001;

ns = not significant.

**Table 3:** Coded and uncoded setting of the process parameters.

Process Parameter	Code	Level				
		
		−2	−1	0	+1	+2
pH	X_1_	6.0	6.5	7.0	7.5	8.0
Temperature/°C	X_2_	40	45	50	55	60
E/S (%)	X_3_	1	1.5	2	2.5	3.0
Reaction time (h)	X_4_	8	9	10	11	12
